# Reports on Hospitals of the United Kingdom

**Published:** 1914-02-28

**Authors:** Henry Burdett


### REPORTS ON
### Hospitals of the United Kingdom.

By SIR HENRY BURDETT, K.C.B., K.C.V.O.

### SERIES III.

### THE ROYAL SURREY COUNTY HOSPITAL, GUILDFORD.

This hospital was established in 1866, and twenty years ago, though it did good work, it presented an aspect of somewhat sleepy administration, whilst the equipment and general appearance of the wards and other parts of the institution were neither smart nor up to date. The Royal Surrey County Hospital in recent years has undergone considerable reconstructions; it now contains a hundred beds, and few more attractive wards can be found than the larger ones in this hospital to-day. Each ward has cross-ventilation, is well equipped, and the choice of colours and general tone of the decorations are both attractive and restful to the eye. It gave us great pleasure to visit this hospital and to observe the good order and smartness which now prevail practically everywhere. The committee would be well advised to supply the whole of the wards with new lockers; the Cromer pattern would not be a bad one to select, and it would be relatively inexpensive. The ventilators in the ceilings of the wards should be closed, and some generous donors found to give a few new pattern combined ward-tables. This is a hospital where much depends upon the quality and efficiency of the resident medical staff. We hope that since the date of our visit arrangements have been made to provide the house surgeon with his own sitting room, and also to give similar accommodation to the two junior resident medical officers. It is always pleasant to see good management in the kitchen and domestic departments, including the stores and food arrangements of a county hospital. It seemed to us that in all these matters the management is strong and exemplary. Taken as a whole this hospital has wonderfully developed in recent years, and has become in fact one of the most interesting of its type and size.

### Structure and Equipment.

We will now deal with the structure and equipment as we found them. With the exception of certain baths, supplied by a great London firm within the last ten years, which have not worn properly and require replacing, the repairs and maintenance throughout are in an exceptionally good state. We are of the opinion that a letter should be addressed to the makers of the defective baths, drawing their attention to the increasingly unsatisfactory appearance of these baths as presented to the visitor, and asking them, for the credit of their firm, to replace them by up-to-date baths of a type that will stand hospital wear and remain in good condition, as they ought to do, for thirty years or more. It struck us that in more than one of the lavatories the atmosphere would be materially improved if the " S " bends were regularly emptied once a month. Such a monthly cleansing and overhauling, of the dates of which a strict record should be kept, would obviate much of the drawback caused by the smallness of some of the slop and soil sinks and other fittings. The operation theatre was attractive and well up to date, but to place the sterilising arrangements on the opposite side of the corridor, facing the theatre, is a bad arrangement for many reasons and should be changed. We observe, too, that the method of handling the coal is defective, owing, we believe, to the absence of proper coal-trolleys, one for each ward, which should be so constructed as to enable them to be readily moved along the corridors and to enter the wards without noise. If the Ipswich type of coal-trolley was introduced it would make the stoking of fires as little noticeable as possible. (See The Hospital, February 21, 1914, in "The Institutional Worker," p. 1.) The nurses are well housed, and most of them have a separate bedroom. We think it would be a good scheme to make a determined effort to furnish and make attractive the nurses' sitting-room and that of the sisters', in such a way as to secure their being used during off-duty hours. The home contains a good supply of baths and other things which must tend to make a nurse's stay at this hospital as comfortable and health-sustaining as possible. The bedroom accommodation of the domestic staff and its equipment is, on the whole, probably as good as, if not better than, that of the majority of British hospitals, quite apart from their size. We are, however, of the opinion that it is a good rule that in any room where more than one servant is sleeping there should be screens in order to secure a maximum of privacy, for attention to such details tends to raise the tone and efficiency of the whole staff of a public institution.

### Two Matters for Consideration.

Two points struck us in connection with the regulations as practised at this hospital. First, the system of visiting by the governors is remarkable. The rules lay down that two house visitors shall be appointed every month, and that they shall visit the hospital three times a week and attend to any matters requiring their attention as, and when, they may arise. We feel that a visit to the hospital, with its attendant duties, every alternate day must prove an onerous duty to the majority of the visitors, whilst these ladies or gentlemen must possess great tact and self-suppression, the exercise of which cannot fail to prove burdensome at times.

Another thing that struck us was that although the mortuary block has been reconstructed and greatly improved, and displays a wise endeavour to carry out the reconstruction in the best possible way, one of the three mortuary chambers should be fitted up as a view-room or chapel, where the friends can see their dead under proper conditions. The selected room should be given an atmosphere calculated to excite reverence in the beholder, and so uphold the best traditions of Christian teaching. It should exhibit the most tender consideration for the feelings of the friends, and of reverence for the dead. A hospital should, for educational reasons, be the centre of the highest efficiency in relation to everything which affects the hygienic nursing and medical treatment of the living, and of the deepest respect for the dead. We welcome the reconstructed mortuary as evidence of a wish on the part of the committee to maintain this high standard, and we have little doubt that they will speedily adopt the simple suggestions we have ventured to make in regard to the mortuary. We had abundant evidence during our visit of the personal interest taken in the welfare of the hospital by the committee and the officers, an interest which has conferred immeasurable benefits upon the Surrey County Hospital, and tends to ensure, as we are convinced it will maintain, the high standard of excellence which is to be found in many portions of this institution.

More Permanent Income Essential.

This hospital, like most county hospitals, owing to its present efficiency and the immense value and importance of the work it does for the whole community, has arrived at a stage in its history when the permanent income should be considerably increased. The more efficient, up to date, and modern a hospital like that at Guildford becomes, the more right has it to expect, nay, to demand, that every single inhabitant of the district it serves should take an interest in its work, and contribute his or her share to its support by giving directly some subscription, however small, every year. Again, all should agree to give a measure of personal service each year in the cause and support of the hospital as an act of praise to the Giver of all Good for the blessings of health which they and those dear to them may enjoy. We should like to see the town of Guildford divided into wards, and the appointment of a Ladies' Association, with the Mayoress or some other lady of position and influence as President. The President could then select and appoint a suitable woman to be chairman of each ward and advise with them as to the selection and appointment of a captain of each street in every ward, the duty of such captains being to give sufficient time each year to call on every householder and to use their best endeavours to persuade as many as possible to become regular subscribers of from 1s. a year (or 1d. a month) upwards. Each captain would undertake to collect the subscriptions from the residents in the street under her charge, and to call for the money once a month, or at such other times as might be determined, so as to suit the convenience of all concerned. We believe that if an organisation of this sort was set on foot in Guildford and the right people agreed to take office and work the scheme, the Guildford Hospital would be kept permanently free from debt, and have enough money besides to equip it throughout and to maintain it in the greatest possible efficiency.

**Some Cottage Hospitals will be reported on next week.**

